# An integrated personalized assistive devices approach to reduce the risk of foot ulcer recurrence in diabetes (DIASSIST): study protocol for a multicenter randomized controlled trial

**DOI:** 10.1186/s13063-023-07635-z

**Published:** 2023-10-12

**Authors:** Lisa E. Vossen, Jaap J. van Netten, Chantal D. Bakker, Heleen A. Berendsen, Tessa E. Busch-Westbroek, Edgar J. G. Peters, Louise W. E. Sabelis, Marcel G. W. Dijkgraaf, Sicco A. Bus

**Affiliations:** 1 Rehabilitation Medicine, Amsterdam UMC Location University of Amsterdam, Meibergdreef 9, Amsterdam, the Netherlands; 2 Rehabilitation & Development, Amsterdam Movement Sciences, Amsterdam, the Netherlands; 3 Department of Rehabilitation Medicine, Máxima Medisch Centrum Veldhoven, de Run 4600, 5504 DB Veldhoven, the Netherlands; 4 Department of Rehabilitation Medicine, Reinier de Graaf Gasthuis Delft, Reinier de Graafweg 5, 2625 AD Delft, the Netherlands; 5 Internal Medicine, Amsterdam UMC Location Vrije Universiteit Amsterdam, Boelelaan 1117, Amsterdam, the Netherlands; 6 Rehabilitation Medicine, Amsterdam UMC Location Vrije Universiteit Amsterdam, Boelelaan 1117, Amsterdam, the Netherlands; 7 Epidemiology and Data Science, Amsterdam UMC Location University of Amsterdam, Meibergdreef 9, Amsterdam, the Netherlands; 8 Methodology, Amsterdam Public Health, Meibergdreef 9, Amsterdam, the Netherlands

**Keywords:** Diabetic foot ulcer, Prevention, Cost–benefit analysis, Adherence, Personalized medicine, Footwear, Assistive devices, Temperature monitoring, Education, Motivational interviewing

## Abstract

**Background:**

Preventing foot ulcers in people with diabetes can increase quality of life and reduce costs. Despite the availability of various interventions to prevent foot ulcers, recurrence rates remain high. We hypothesize that a multimodal treatment approach incorporating various footwear, self-management, and education interventions that matches an individual person’s needs can reduce the risk of ulcer recurrence with beneficial cost-utility. The aim of this study is to assess the effect on foot ulcer recurrence, footwear adherence, and cost-utility of an integrated personalized assistive devices approach in high-risk people with diabetes.

**Methods:**

In a parallel-group multicenter randomized controlled trial, 126 adult participants with diabetes mellitus type 1 or 2, loss of protective sensation based on the presence of peripheral neuropathy, a healed plantar foot ulcer in the preceding 4 years, and possession of any type of custom-made footwear will be included. Participants will be randomly assigned to either enhanced therapy or usual care. Enhanced therapy consists of usual care and additionally a personalized treatment approach including pressure-optimized custom-made footwear, pressure-optimized custom-made footwear for indoor use, at-home daily foot temperature monitoring, and structured education, which includes motivational interviewing and personalized feedback on adherence and self-care. Participants will be followed for 12 months. Assessments include barefoot and in-shoe plantar pressure measurements; questionnaires concerning quality of life, costs, disease, and self-care knowledge; physical activity and footwear use monitoring; and clinical monitoring for foot ulcer outcomes. The study is powered for 3 primary outcomes: foot ulcer recurrence, footwear adherence, and cost-utility, the primary clinical, patient-related, and health-economic outcome respectively.

**Discussion:**

This is the first study to integrate multiple interventions for ulcer prevention into a personalized state-of-the-art treatment approach and assess their combined efficacy in a randomized controlled trial in people with diabetes at high ulcer risk. Proven effectiveness, usability, and cost-utility will facilitate implementation in healthcare, improve the quality of life of high-risk people with diabetes, and reduce treatment costs.

**Trial registration:**

ClinicalTrials.gov NCT05236660. Registered on 11 February 2022.

**Supplementary Information:**

The online version contains supplementary material available at 10.1186/s13063-023-07635-z.

## Administrative information

Note: the numbers in curly brackets in this protocol refer to SPIRIT checklist item numbers. The order of the items has been modified to group similar items (see http://www.equator-network.org/reporting-guidelines/spirit-2013-statement-defining-standard-protocol-items-for-clinical-trials/).

**Table:** 

Title {1}	An integrated personalized assistive devices approach to reduce the risk of foot ulcer recurrence in diabetes (DIASSIST): study protocol for a multicenter randomized controlled trial
Trial registration {2a and 2b}.	clinicaltrials.gov: NCT05236660. Registered on 11 February 2022.
Protocol version {3}	Protocol version 2, dated: 25–11-2022
Funding {4}	The DIASSIST trial is funded by ZonMw (the Netherlands Organization for Health Research and Development, project nr. 853,001,116), and co-funded by OFOM (the Dutch Foundation for the Development of Orthopedic Footwear). The funders had no influence on the study design; the collection, management, analysis, and interpretation of data; writing of the report; and the decision to submit the report for publication, and had no ultimate authority over any of these activities.
Author details {5a}	^1^ Amsterdam UMC location University of Amsterdam, Rehabilitation Medicine, Meibergdreef 9, Amsterdam, the Netherlands ^2^ Amsterdam Movement Sciences, Rehabilitation & Development, Amsterdam, the Netherlands ^3^ Máxima Medisch Centrum Veldhoven, Department of Rehabilitation Medicine, de Run 4600, 5504 DB, Veldhoven, the Netherlands ^4^ Reinier de Graaf Gasthuis Delft, Department of Rehabilitation Medicine, Reinier de Graafweg 5, 2625 AD, Delft, the Netherlands ^5^ Amsterdam UMC location Vrije Universiteit Amsterdam, Internal Medicine, Boelelaan 1117, Amsterdam, the Netherlands ^6^ Amsterdam UMC location Vrije Universiteit Amsterdam, Rehabilitation Medicine, Boelelaan 1117, Amsterdam, the Netherlands ^7^ Amsterdam UMC location University of Amsterdam, Epidemiology and Data Science, Meibergdreef 9, Amsterdam, the Netherlands ^8^ Amsterdam Public Health, Methodology, Meibergdreef 9, Amsterdam, the Netherlands
Name and contact information for the trial sponsor {5b}	Amsterdam UMC, University of Amsterdam.
Role of sponsor {5c}	This study was initiated by investigators of Amsterdam UMC, University of Amsterdam. The investigators are involved in all aspects of the study design, writing, and the decision to submit the report for publication.

## Introduction

### Background and rationale {6a}

Globally, every 30 s, a lower limb is lost due to diabetes [1]. Most amputations are preceded by a foot ulcer with an annual incidence rate of 2.2% and a lifetime risk of 19–34% in persons with diabetes [2, 3]. In particular, the risk for ulcer recurrence is high: 40% within 1 year after healing [2]. Ulcers and amputations are key outcomes of diabetic foot disease, which ranks 10th in leading causes of global disease burden [4] due to the significant negative impact on quality of life and patient mobility [5]. Furthermore, the treatment of these foot ulcers is costly, among others due to high risk of infection which often leads to hospitalization and possibly amputation. This amounts to an average cost of about €10,000 per ulcer episode [6]. Ulcer prevention is an important means to decrease this patient and healthcare burden [2].

Approximately half of all foot ulcers are located on the plantar foot surface, and are caused by a combination of risk factors of which repetitive mechanical stress on the plantar foot surface in the presence of loss of protective sensation due to peripheral neuropathy is the most prevalent [2]. To prevent plantar foot ulcer recurrence, current guidelines recommend an approach consisting of multiple modalities, including (1) custom-made footwear with a demonstrated plantar pressure-relieving effect that is constantly worn by the patient, both outdoors and indoors; (2) regular foot inspection and care; (3) instructing patients to perform self-management by monitoring their own foot temperature; and (4) patient and family education [7]. While all these modalities can, theoretically, be offered to individuals with diabetic foot disease as part of usual care, a combined and integrated prescription is unique and, to the best of our knowledge, currently not implemented anywhere globally.

#### Footwear and adherence

Repetitive, relatively high mechanical stresses on the plantar foot during weight-bearing activity are a common cause of plantar foot ulcers [2]. Because of neuropathy, this process often goes unnoticed by the patient, which prevents timely intervention and can thus lead to ulceration. The key intervention to reduce these pressures is (custom-made) footwear. Such footwear is recommended based on studies demonstrating its positive effects in protecting the feet of people with diabetes [8]. Based on guideline recommendations and scientific evidence from interventional and observational studies, a state-of-the-art protocol to design and optimize (custom-made) footwear for pressure relief in people with diabetes has been recently developed [9]. This protocol includes a matrix containing footwear and insole design features for different domains of foot pathology and a 10-step algorithm for optimizing pressure relief that incorporates in-shoe pressure measurements for footwear evaluation. The protocol was designed to facilitate consistency in the prescription, design, and manufacturing of adequate footwear, to help improve ulcer prevention. However, the effects of this protocol have not yet been investigated.

Footwear with pressure-improved capabilities needs to be worn to be effective. Previous research has shown a statistically non-significant relative risk reduction of 11% in foot ulcer recurrence for pressure-improved custom-made footwear in people at high risk of plantar foot ulceration, which increased to a statistically significant 46% when footwear was worn as recommended [10]. Footwear adherence, expressed as the number of steps taken in prescribed footwear as a percentage of the total number of steps taken, in people with diabetes at high ulcer risk is on average ~ 70% [11]. Adherence to wearing prescription custom-made footwear is significantly lower inside the house than away from home [11]. A survey shows this lower adherence inside the house to be related to the perception of the footwear being heavy, difficult to don and doff, warm and dirty, or simply out of habit [12]. Additionally, if people at high ulcer risk perceive their footwear as less beneficial, footwear is worn less frequently [11]. Pressure-relieving custom-made footwear that is specifically designed for indoor use and that is lighter and easier to don and doff compared to conventional custom-made footwear has recently been developed [13]. In a non-controlled study, indoor adherence improved significantly at 1 and 12 months after the provision of such indoor footwear [14]. A controlled prospective follow-up study in a larger study population is necessary to confirm these findings and to investigate the effects on ulcer recurrence rates.

#### Self-care and self-management

Another cornerstone of ulcer prevention is regular foot inspection, self-care, and self-management. Current guidelines recommend patients to check and monitor their feet and footwear on a daily basis [7]. If patients are adherent, fewer ulcers recur [15]. To enhance regular foot inspection, daily monitoring of contralateral differences in foot temperature is a self-management strategy recommended in current guidelines [7], based on the outcomes of multiple randomized controlled trials (RCTs) [16–20]. Temperature differences are not seen or felt because of sensory neuropathy, but can easily be measured and monitored by patients themselves using a thermometer. A local increase in foot temperature, i.e., a “hotspot,” can be a preliminary sign of inflammation preceding ulceration [16–18, 20]. A meta-analysis of RCTs shows that daily monitoring of foot temperature reduces the risk of foot ulcers by around 25% [21]. Key in this self-management strategy is that the signaling of a hotspot is followed by taking the pressure off the foot with decreasing ambulatory activity; adherence to this part of the strategy resulted in a significantly higher ulcer risk reduction [20]. However, adherence to daily monitoring of foot temperatures has shown to be low in almost 40% of study participants, and only around 25% of participants who measured a hotspot decreased their ambulatory activity [20]. Daily self-management for ulcer prevention is often seen as a burden, and daily thermometry adds to that burden [22], explaining some of the low adherence. And while daily foot temperature monitoring is a promising prevention method, to date the method is hardly implemented in clinical practice. Adjustments in the intervention seem to be needed to improve usability and adherence and decrease the patient burden, for example by personalizing the measurement locations [20]. The effects of these adjustments need to be investigated.

#### Patient and family education

A final cornerstone of ulcer prevention is structured patient and family education [7], to inform and educate people with diabetes who are at risk about recommended self-care behaviors. However, in clinical practice, this education is non-structured and non-personalized. Furthermore, studies show only minor-to-none clinical effects of education [15]. This may be the result of education being offered at a single moment only, and not including behavior-change techniques.

The effect of education that is provided in a structured manner and repeated over time is unknown. There is currently no state-of-the-art education protocol available that could be implemented in clinical practice. More well-designed studies are needed to investigate the effects of different education techniques that are offered more frequently and in a structured manner [8]. Additionally, education should be personalized by taking into account patient preferences and existing knowledge, adherence to footwear, self-care, and self-management and focusing on behavior change [15].

Adherence and behavior change are important in all preventative interventions described above. Significantly better outcomes are reported when patients are adherent to their prescribed treatments [10, 15, 20], suggesting that supporting patients in changing their behavior towards higher adherence will contribute to ulcer prevention. A behavior change intervention such as motivational interviewing has shown to be effective in changing behavior in multiple domains, including diabetes care [23, 24]. It is a method focusing on increasing the intrinsic motivation of the patient by exploring the patient’s own reasons for change and solving the ambivalence against change [25]. The effect of motivational interviewing on treatment adherence in patients with diabetes who are at high risk for foot ulceration seems promising, but needs further investigation in a larger population [25, 26].

#### High ulcer recurrence rates

Despite multiple studies showing positive effects and various treatment approaches recommended for foot ulcer prevention in guidelines, ulcer recurrence rates remain high in clinical practice [2]. Several potential explanations can be given. First, the abovementioned recommendations are not or insufficiently implemented [27]. Lack of insight in the costs and cumulative effectiveness of these modalities may play a role here, as these interventions have only been studied as a single modality [8, 28], not from a multimodal treatment perspective, and cost-utility or cost-effectiveness of these interventions is unknown [7]. Second, even when implemented, the interventions are often not state-of-the-art according to the most recent available evidence. Third, lack of or lower patient adherence reduces the effect of preventative interventions. Improvement in this treatment adherence will positively affect the effectiveness of these interventions. New approaches are needed to address these challenges and resolve the barriers found, so as to better help prevent ulcer recurrence in people with diabetes.

#### Personalized treatment approach

The approach with the largest potential of helping prevent foot ulcer recurrence combines evidence-based state-of-the-art interventions as described above, integratively addressing all modalities and using a personalized approach to match an individual’s needs and preferences [27]. Such an approach requires (financial) investments. The design of pressure-optimized custom-made footwear requires investments in equipment, training of personnel, and measurement time, none of which are currently reimbursed through healthcare insurers. The provision of custom-made indoor footwear adds to the costs, as do tools to monitor footwear use and to measure at-home foot temperatures, and structured education like motivational interviewing. At-home foot temperature monitoring also requires daily assessment by the patient and possibly further diagnosis and foot care by a healthcare professional when hotspots are found. The risk of false-positive outcomes may result in over-diagnosis and treatment, adding burden to the patient and healthcare system. Finally, a multimodal state-of-the-art treatment approach likely changes the organization of care to one where more scientific, data-driven care is provided. This requires intensified collaboration within the multidisciplinary team, intensified patient monitoring, and more personalized treatment decisions based on objective data, all potentially increasing costs.

However, with the treatment of a single foot ulcer costing on average €10,000 and the reduction in quality of life of patients when having a foot ulcer [5, 6], we hypothesize that the investments required for an integrated personalized approach will be offset my substantial savings in ulcer treatment costs and thus not increase total costs for treating these people with diabetes who are at high risk of ulceration. To test this hypothesis, the cost-utility of an integrated personalized treatment approach for ulcer prevention needs to be investigated. The Diabetic Foot Assistive Devices Trial (DIASSIST) aims to assess the clinical effectiveness, patient adherence, and cost-utility of such an integrated personalized assistive devices approach to reduce the risk of foot ulcer recurrence in people with diabetes mellitus.

### Objectives {7}

#### Primary objectives

The primary objectives are to assess the effects of an integrated personalized assistive devices approach in people with diabetes who are at high risk of ulceration on foot ulcer recurrence, adherence to wearing custom-made footwear, and cost-utility. These objectives cover three different domains of outcome measures: clinical, patient-related, and health-economic, respectively.

#### Secondary objectives

The secondary objectives are to assess the effects of an integrated personalized assistive devices approach in people with diabetes who are at high risk of ulceration on foot ulcer recurrence on the plantar surface and at high-risk locations; ulcer-free survival days; falls; wearing time of prescribed footwear; in-shoe peak plantar pressure; cumulative plantar tissue stress; footwear wear-and-tear; ulcer risk prediction score; treatment and footwear satisfaction; foot-related self-care; knowledge of foot care; capabilities, opportunities, and motivations to perform recommended footwear behavior (COM-b) score; quality of life; physical activity; costs; quality-adjusted life years; and cost-effectiveness. For participants who develop a foot ulcer, secondary outcomes further include time to ulceration, ulcer severity, time to healing, and referral time.

#### Hypotheses

We hypothesize that an integrated personalized assistive devices approach in addition to usual care will significantly lower the proportion of people with foot ulcer recurrence, significantly increase adherence to wearing custom-made footwear, and save costs per quality-adjusted life year gained, compared to usual care alone in people with diabetes at high risk of foot ulceration. These hypotheses are based on the superiority of the intervention compared to usual care, studied in a multicenter RCT.

### Trial design {8}

The study design is a multicenter, parallel-group, superiority RCT with two study arms with a 1:1 allocation ratio:

- Enhanced therapy, which includes usual care as provided in the Netherlands and, in addition, an integrated personalized assistive devices approach
- Usual care as provided in the Netherlands

## Methods: participants, interventions, and outcomes

### Study setting {9}

Recruitment will take place from one university and two community-based hospitals with a multidisciplinary diabetic foot clinic in different regions in the Netherlands, and from professional practices of podiatrists who participate in these multidisciplinary foot clinics. Each diabetic foot clinic and the orthopedic shoe company that is contracted by the clinic will operate as one of the study centers where all the study assessments take place. Within each center, a physician in rehabilitation medicine, a podiatrist, and an orthopedic shoe technician will be involved. The participating hospitals are Amsterdam UMC (location AMC and location VUmc), Máxima Medisch Centrum (Veldhoven), and Reinier de Graaf Gasthuis (Delft). All centers have successfully participated in the recruitment and follow-up of people with diabetes at high ulcer risk in previous trials [10, 11, 29]. Patient education in the enhanced therapy group, including motivational interviewing, takes place via telephone.

### Eligibility criteria {10}

#### Population

The study population will consist of people with diabetes who are considered at high risk for developing a foot ulcer, i.e., risk grade 3 of the International Working Group on the Diabetic Foot stratification scheme [30].

#### Inclusion criteria

In order to be eligible to participate in this study, a participant must meet all of the following criteria:

- Diabetes mellitus type 1 or 2
- Age 18 years or above
- Loss of protective sensation based on the presence of peripheral neuropathy [31]
- A healed plantar foot ulcer or amputation of a part of the foot in the preceding 4 years until 2 weeks before study inclusion
- In possession of custom-made (orthopedic) footwear, which is either fully custom-made (“Orthopedic footwear type A” or OSA) or custom-made insoles worn in extra-depth shoes (“Orthopedic footwear type B” or OSB), or Orthopedic Provisions to off-the-shelf Footwear (OVAC), according to the Dutch healthcare system
- Ability to provide informed consent

#### Exclusion criteria

A subject who meets any of the following criteria will be excluded from participation in this study:

- Foot ulcer or open amputation site(s)
- Active Charcot’s neuroarthropathy
- Foot infection, based on criteria of the PEDIS classification [32]
- Amputation proximal to the metatarsal bones in both feet
- Healed ulcer on the apex of digitus 2–5 as the only ulcer location in the past 4 years, as surgical intervention by flexor tenotomy is a more likely and guideline-recommended treatment for people with such a history [7, 30], rather than the enhanced therapy under investigation
- Severe illness that would make 12 months’ survival unlikely, based on the clinical judgment of the physician
- Concomitant severe physical or mental conditions that limit the ability to follow instructions for the study, based on the clinical judgment of the physician

### Who will take informed consent? {26a}

After careful initial assessment of the eligibility of participants based on the inclusion and exclusion criteria, potentially eligible participants will be informed about the study by their physician or podiatrist. The participant will be given 1 week to consider participation and will then be contacted by the investigator about their willingness to participate in the study. Informed consent will be obtained prior to the start of the first assessment during the baseline study visit.

### Additional consent provisions for collection and use of participant data and biological specimens {26b}

The informed consent includes an optional choice for participants whether or not their data may be used in future studies. No biological specimens will be collected during this trial.

### Interventions

#### Explanation for the choice of comparators {6b}

Participants will be randomly assigned to either enhanced therapy or usual care. The comparison to usual care was chosen as being the current standard of care in the Netherlands of which participants cannot be deprived for ethical reasons. Enhanced care includes the intervention under investigation in addition to usual care, as it is key to prove that enhanced therapy is superior to usual care. Usual care consists of care as provided to high-risk people with diabetes in the Netherlands, following national and international guidelines and standards [33–35]. This includes foot care and screening by a podiatrist, a contracted diabetes pedicure, and if needed, a multidisciplinary team, every 1–3 months [33]. It also includes custom-made footwear that may or may not already be evaluated using in-shoe plantar pressure assessment as usual care in some centers. Usual care may thus include one or more of the enhanced therapy interventions, although in usual care these interventions are mostly offered in a less structured way, not according to a state-of-the-art protocol, not as an integrated approach, and without much personalization to the approach.

#### Intervention description {11a}

The treatment of participants who are randomized to enhanced therapy will consist of usual care, and in addition a personalized state-of-the-art integrated assistive devices approach that consists of:

A. Custom-made footwear, evaluated and optimized using in-shoe pressure analysis, and re-evaluated after 6 months
B. Custom-made footwear for specific use indoors, also evaluated and pressure-optimized, and re-evaluated after 6 month
C. At-home daily foot temperature monitoring. Personalized to high-risk regions
D. Personalized and structured education consisting of a structured visual model to explain risk factors and treatments, quantitative feedback on in-shoe pressures, temperature measurements and footwear use, and motivational interviewing to help improve or sustain device use

**Fig. 1:**
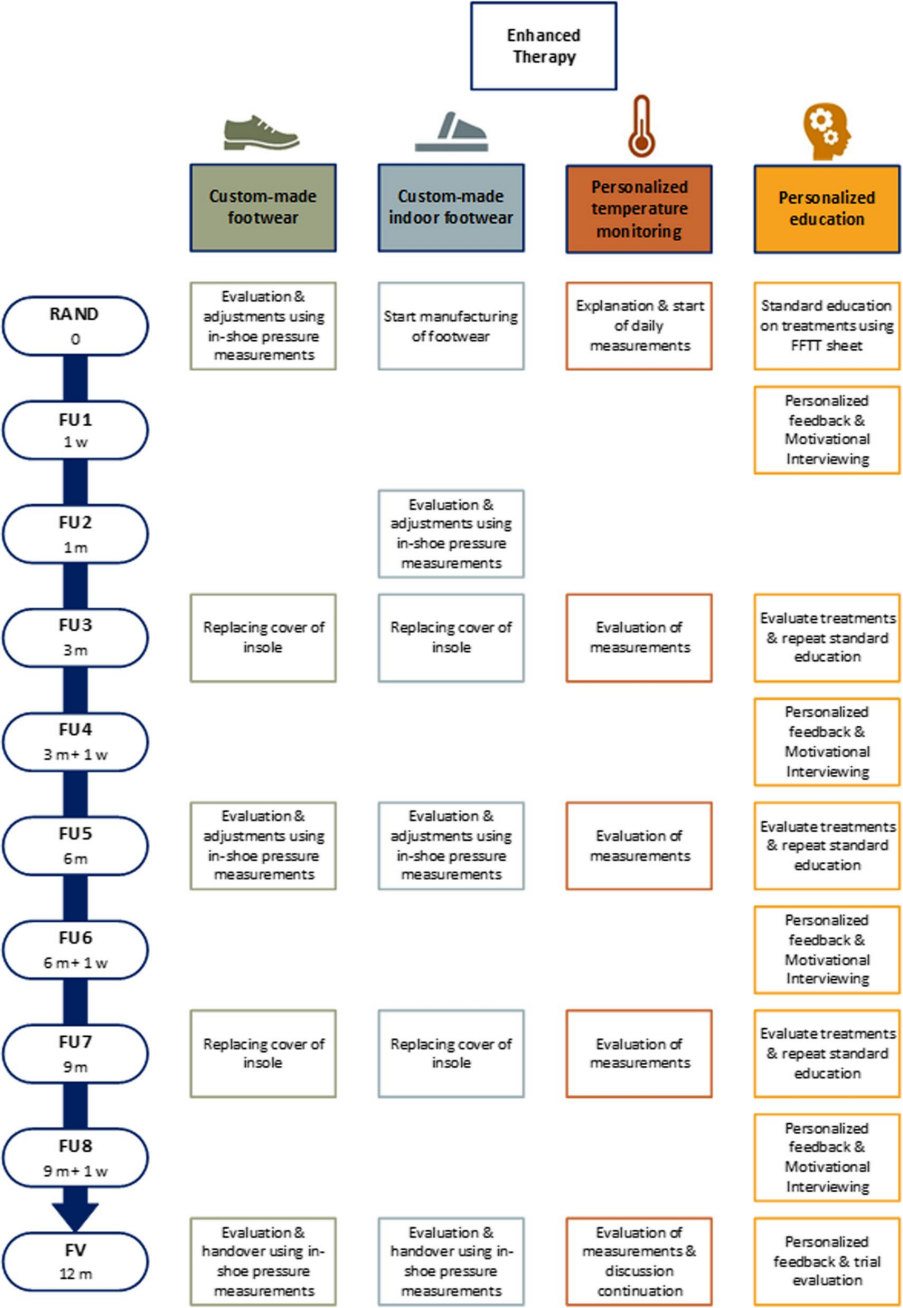
Schedule of intervention modalities. All follow-up visits can deviate a maximum 2.5 weeks from the scheduled timepoint. RAND, randomization visit; FU_x_, follow-up visit (numbered by visit number); UV, unscheduled visit; FV, final visit

The intervention in this RCT is denoted as “enhanced therapy,” with the corresponding timeline depicted in Fig. 1. A prescription of this multimodal approach is considered the most comprehensive preventative treatment approach in accordance with international guidelines, but currently not available or implemented in the Netherlands or, as far as we are aware, anywhere globally. Moreover, in the current study, the interventions are provided as state-of-the-art approaches, following the latest evidence for design, assessment, manufacturing, and use. Finally, the four modalities used are offered in a personalized manner, to match the individual participant’s situation and their personal needs.

A. Custom-made footwear

At the start of the inclusion, each participant possesses custom-made footwear, as defined in the inclusion criteria, that will be assessed on shoe and insole design features. In the enhanced therapy group, this footwear will be checked against the latest standards, as described in the state-of-the-art design protocol for custom-made footwear [9]. As part of this protocol, the footwear will be evaluated during walking using in-shoe plantar pressure analysis (Pedar-X system, Novel GmbH, Munich, Germany). Footwear will be targeted for pressure improvement if peak pressures are > 200 kPa or if the research team and shoe technician believe a more optimal pressure result is feasible. The aim is to reduce peak pressure with 25% compared to baseline or to an absolute level below 200 kPa. Every 3 months, the top layer of the insoles will be replaced, as previous research showed this to be a key pressure-reducing intervention, likely because of wear and tear of the top cover [36]. After 6 months, the custom-made footwear will be re-evaluated using in-shoe plantar pressure measurements and modified if needed, following the same protocol as described above if peak pressures are > 200 kPa or improvement is considered feasible by the team.

B.  Custom-made indoor footwear

Alongside the regular custom-made footwear that participants already have, custom-made footwear specifically for indoor use will be designed and manufactured [13]. The manufacturing procedure will be explained to participants, and the potential benefits and harms will be discussed. While recommended to have the indoor footwear prescribed, participants are free to accept or decline the custom-made indoor footwear. If a participant declines custom-made indoor footwear, the option will again be discussed in 3 months. If a participant then decides to have custom-made indoor footwear prescribed, the above procedures will be followed.

The custom-made indoor footwear will be designed and manufactured using the last of the regular custom-made footwear [13]. The outsole and insole of the shoe (i.e., all parts of the footwear plantar to the foot) will be designed and manufactured to be similar to the regular custom-made footwear. For the footwear upper, light-weight easy-to-apply materials will be used and the footwear will be made in such a way that it facilitates donning and doffing and appears as indoor footwear, so to prevent the use of this footwear outdoors. The custom-made indoor footwear will be assured to have the same pressure-relieving properties as the regular custom-made footwear, evaluated and guided by in-shoe plantar pressure measurements [9]. We allow a maximum 10% higher peak pressures for the indoor footwear compared to the regular custom-made footwear, because of lower walking speeds and thus lower peak pressures, normally executed in the home [37]. Production will generally take 6 weeks. Follow-up measurements, footwear adjustments, and improvement will be according to the same protocol as for the regular custom-made footwear.

C. Personalized temperature monitoring

At-home daily foot temperature monitoring will be conducted using infrared handheld thermometry (TempTouch®, MR3 Health, San Antonio, TX, USA) [20, 38]. The thermometer has a goose-neck design that helps the user to reach any point on the bottom of the foot. The thermometer is equipped with a “touch sensor” tip that detects contact with the skin. Thus, to operate the device, the user places the tip of the device on the skin, which then automatically triggers a temperature measurement and displays it on a liquid crystal display screen in °C. To standardize training, instructions on the correct use of the thermometer are provided both verbally and on paper.

Participants will measure at three high-risk plantar regions per foot, including the previous ulcer location and two other regions where high in-shoe peak pressures are present, or where a pre-ulcerative lesion is present. The number of measurement sites can deviate from three if fewer or more high-risk regions are defined for a participant, as part of the personalized approach. A maximum of four high-risk regions is allowed to ensure practical feasibility and minimize treatment burden for participants. Foot temperature is measured once per day, preferably in the morning directly after getting out of bed. To facilitate measurement and assure adherence, the participant is advised to place the thermometer on a bedside table. If skin temperature measured in a region is ≥ 2.2°C higher than in the corresponding region on the contralateral foot, the temperatures are recorded in a logbook. If this above-threshold temperature difference is present for two consecutive days, a “hotspot” is present, and the participant is instructed to contact the research team. The research team will ask the participant about any swelling, change in color, change in structure, or drainage present at the hotspot and the use of their prescribed footwear. Based on these outcomes, the investigator may refer the participant for further diagnosis and foot care to their podiatrist. In any case, the participant will be instructed to decrease ambulatory activity with approximately 50% until the temperatures normalize (< 2.2°C temperature difference). If applicable, the participant will also be advised to increase the use of their custom-made footwear. If the temperature difference is larger than 4°C, or if temperatures do not normalize and are above threshold for four consecutive days, the participant will be referred immediately by the podiatrist. If needed, direct referral for treatment to specialized care will take place. This may involve, among other things, immobilization of the foot.

During the first 2 weeks, participants will measure foot temperatures daily and record findings in a logbook. This run-in period is used for participants to become acquainted to the measurements and for the researchers to receive indications of the participant’s fluctuations in foot temperature over a 2-week period. After this run-in period, participants will still measure temperatures daily, but the participant can choose whether or not temperatures are recorded in the logbook. When an above-threshold temperature difference is found, participants are instructed to record these instances in the logbook.

At each study visit, participant experiences with and adherence to the at-home foot temperature measurements will be discussed, as well as the added value based on the frequency that hotspots are found. If the participant reports the temperature measurements to be a burden and considers to discontinue measurements, alternative solutions to personalize the measurement protocol and lower the burden will be discussed and if needed implemented. The researchers may also decide it is no longer of value to continue the temperature measurements with a participant, for example when a hotspot is never measured. Both outcomes contribute to the personalization of treatments that is key in this study.

D. Personalized education

Throughout the intervention, participants will receive structured education during their study visits provided by the investigator. The education will be structured using the “Fragile Feet – Trivial Trauma” (FFTT) model [39]. The model will be personalized, to reflect the situation of the individual participant, based on the presence or absence of risk factors as obtained during baseline screening. The personalized FFTT model will be provided to the participant on a printed leaflet and will be used to guide the participant through an explanation of multiple risk factors and the intervention modalities that reduce these risks. A collaborative approach in discussing these interventions will be taken [40], in a first-person narrative [41]. Participants will be asked to explain the leaflet in their own words to check for their understanding, to allow for teach-back [42].

Following randomization, four specific follow-ups are scheduled for patient education that includes motivational interviewing, and will be conducted by one of the senior investigators who are trained in motivational interviewing methods by an expert psychologist (FU, see Fig. 1). The first follow-up is by telephone, 1 week after randomization. When participants are non-adherent to wearing their custom-made footwear, defined as an average wearing time < 8 h/day, the education will focus on changing behavior to increase adherence, using motivational interviewing (see below). For participants who are adherent to wearing their custom-made footwear, the education will focus on retaining this desired behavior. The next three follow-ups will take place 1 week after each regular 3-monthly study visit and will also be conducted by telephone. For these follow-up interviews, the personalized FFTT model will be updated with outcomes of in-shoe pressure, physical activity, and wearing time measurements, collected at each regular study visit. These outcomes will be discussed during the interviews, together with the self-care behavior of the participant. All follow-ups for education will contain motivational interviewing, that is provided in accordance with a protocol developed for the study and based on published protocols on motivational interviewing to improve footwear adherence [26]. If adherence to footwear use, temperature monitoring, or self-care is high, motivational interviewing will focus on retaining this desired behavior; if adherence is low, motivational interviewing will focus on behavior change. All telephone conversations will be recorded and a random subset will be analyzed for treatment fidelity, scored using the Motivational Interviewing Treatment Integrity (MITI 4) code [43].

#### Criteria for discontinuing or modifying allocated interventions {11b}

Subjects can leave the study at any time for any reason if they wish to do so without any consequences. The investigator or treating physician can decide to withdraw a subject from the study for urgent medical reasons. All interventions are personalized and can change during the follow-up period as described in the previous sections.

#### Strategies to improve adherence to interventions {11c}

Adherence is one of the primary study outcomes and thus a major focus of attention. Strategies to improve adherence include:

- Provision of pressure-optimized custom-made footwear for indoor use, to improve footwear adherence indoors
- Education, to emphasize the importance of the intervention
- Personalization of treatments, to provide treatments specifically focused and tailored towards the participant, avoiding unnecessary procedures
- Diaries, to remind participants of their checks and tasks
- Follow-up calls, to give feedback about measurements and adherence
- Motivational interviewing, to motivate participants to improve low adherence and retain high adherence

Procedures for monitoring adherence include:

- Sensors (OrthoTimer; Rollerwerk, Balingen, Germany) in the participant’s custom-made (indoor) footwear, to monitor wearing time of the custom-made (indoor) footwear
- Measuring physical activity levels with an activity monitor (MoveMonitor; McRoberts, the Hague, the Netherlands), to measure weight-bearing time and number of steps taken. Combined with measurements of wearing time, this generates footwear adherence.
- Diaries and questionnaires; to check adherence by self-reporting

#### Relevant concomitant care permitted or prohibited during the trial {11d}

All concomitant care and interventions are permitted during the trial, as long as participants meet the conditions for in- and exclusion at the time of taking informed consent.

#### Provisions for post-trial care {30}

After the participant completes the follow-up period, in-shoe pressure measurements done at the final study visit are shared with the shoe technician and physician as post-trial care. All participants receive a feedback sheet with information on measurements and outcomes throughout the study period. Participants from the enhanced therapy group may, if preferred, continue at-home foot temperature measurements. When the primary study outcomes are available after the last visit of the last participant, study results will be shared with all participants in writing.

### Outcomes {12}

There are three primary study outcomes in this study, which are:

- Foot ulcer recurrence during the 12-month follow-up
- Adherence to wearing custom-made footwear
- The cost (savings) per quality-adjusted life year (QALY) gained (i.e., cost-utility)

These represent a clinical, patient-related, and health-economic perspective, respectively. Foot ulcer recurrence is defined as “a break of the skin of the foot that involves as a minimum the epidermis and part of the dermis, in a person who has a history of foot ulceration, irrespective of location and time since the previous foot ulcer” [15], is monitored during the entire study period, and is—on group level—calculated as the proportion of participants with one or more foot ulcers during the study period. Footwear adherence is defined as the percentage of steps taken in prescribed footwear, calculated by combining physical activity and wearing time measurements [44]. It is calculated over 1 week, at baseline and (for outcomes) at 6 and 12 months’ follow-up. Cost-utility is monitored during the entire study period, costs are summed throughout the 12-month period [45], and utility is scored with the EQ-5D-5L questionnaire every 3 months [46].

**Fig. 2:**
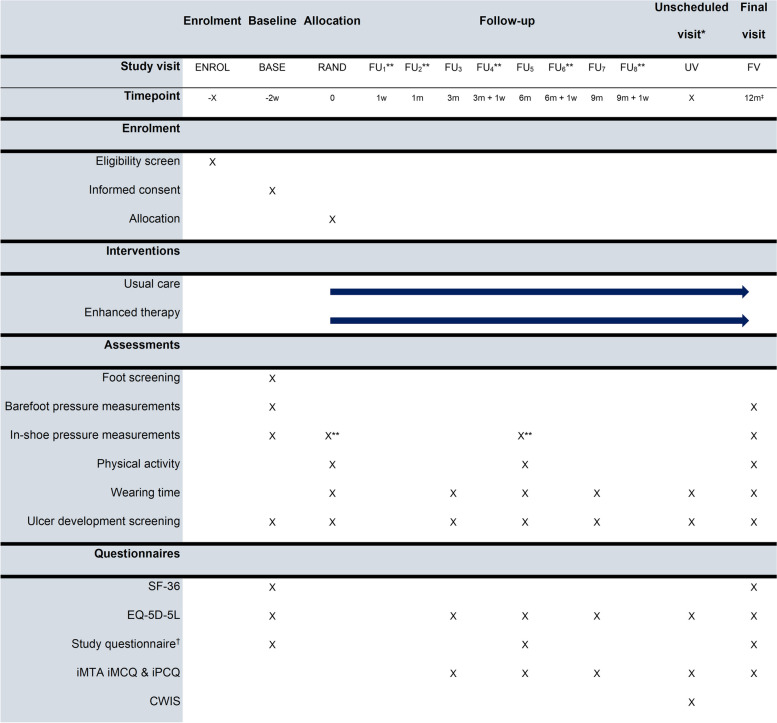
Study design and main procedures for participants. All follow-up visits can deviate a maximum 2.5 weeks from the scheduled timepoint. * = An unscheduled visit becomes a scheduled visit when a participant develops an ulcer. This visit will be repeated every 3 months if an ulcer is not healed, which is the same frequency as normal follow-up visits. ‡ = Regular final visit will be at 12 months. However, if a participant has an ulcer at 12 months, there will be extra follow-up visits after the final visit. These will take place at 15 and 18 months or, if the ulcer has healed earlier, at 2 weeks after healing. The extra follow-ups follow the procedures of the unscheduled visit. ** = For enhanced therapy group only. † = the study questionnaire consists of a combination of multiple validated questionnaires, including adapted versions of the Monitor Orthopedic Footwear, treatment satisfaction questionnaire, COM-b (Capabilities, Opportunities, Motivations) questionnaire, and an assessment of knowledge about foot care based on the PIN questionnaire (Patients’ Interpretation of Neuropathy). ENROL, enrolment visit; BASE, baseline visit; RAND, randomization visit; FU_x_, follow-up visit (numbered by visit number); UV, unscheduled visit; FV, final visit; SF-36, quality of life questionnaire; EQ-5D-5L, quality of life questionnaire measuring quality-adjusted life years; iMTA, Institute for Medical Technology Assessment questionnaires on medical consumption and productivity costs; CWIS, Cardiff Wound Impact Schedule questionnaire

Secondary study outcomes are as follows: proportion of participants with one or more recurrent foot ulcers on the plantar side of the foot [15]; proportion of participants with one or more recurrent foot ulcers at the predefined personalized high-risk locations [15]; ulcer-free survival days [47]; falls [48]; wearing time of custom-made footwear [49]; in-shoe peak plantar pressure [47]; cumulative plantar tissue stress [44]; footwear wear-and-tear [50]; ulcer risk prediction score [29]; treatment and footwear satisfaction [51]; foot-related self-care [52]; knowledge of foot care [53]; capabilities, opportunities, and motivations (COM-b) score [54]; quality of life (in participants with [55, 56] or without [5] a recurrent foot ulcer during the trial); physical activity [57]; costs [45]; QALYs [46]; and cost-effectiveness [45]. In participants with an ulcer, other secondary outcomes are as follows: time to ulceration [58], ulcer severity [59–61], time to healing [58], and referral time [62]. Definitions for all secondary outcomes are as provided in the reference following each secondary outcome. All secondary outcomes are monitored during the entire study period using assessments and questionnaires during follow-up visits (see Fig. 2).

### Participant timeline {13}

Participants who consent to participate and who meet the in- and exclusion criteria will have a baseline visit for patient screening, physical examination, and other measurements (details provided in the “Data collection and management” section). In a next visit, participants will be randomly assigned to the enhanced therapy or usual care group. Each participant will be followed for 12 months or, in case of a foot ulcer present at the 12 months’ follow-up visit, up to a maximum of 18 months, the latter 6 months for foot ulcer monitoring only. Figure 2 shows an overview of the study design and participant timeline.

### Sample size {14}

Based on an analysis of RCTs that have recurrent foot ulcers in people with diabetes as outcome, a risk reduction in participants with one or more recurrent foot ulcers up to 75% can be expected from a state-of-the-art multimodal treatment approach [63]. Using a more conservative estimate, we expect a risk reduction of 59% for the enhanced therapy group compared to usual care. This is based on previous studies that show that footwear and at-home foot temperature monitoring interventions combined can achieve such effect sizes [2, 15, 63]. With an expected ulcer recurrence in 37% of the participants in usual care [2, 20], this 59% risk reduction leads to a hypothesized 15% ulcer recurrence rate in the enhanced therapy group. With 15% ulcer recurrence in enhanced therapy and 37% in usual care, *α* = 0.05 (two-sided) and power = 80%, enrolment ratio of 1, power calculations show that 122 participants are required (61 per study group). We expect no dropout or missing data for the intention-to-treat analysis of the clinical outcome based on previous trial experience that clinical outcome can be obtained in each participant [20], which means that a total 122 participants are required for the primary clinical outcome.

For footwear adherence as the primary patient-related outcome measure, we expect 71% (sd 25%) adherence in usual care at 12 months based on findings from previous research [10, 12]. We expect an increase in footwear adherence in the enhanced therapy group to a level of 85% at 12 months, through the effect of indoor footwear provision [12] and structured education. With *α* = 0.05 (two-sided) and power = 80%, enrolment ratio of 1, power calculations show that 100 participants are required (50 per study group). Including a drop-out rate of 20%, this leads to a sample size of 120 participants in total for the primary patient-related outcome.

For cost-utility as primary health-economic outcome measure, we anticipate a clinically relevant 0.1 gain in QALYs for the enhanced therapy group with group standard deviations for QALYs of 0.15 [64]. We use an estimation of cost difference of €0 per participant between both groups with group standard deviations for costs of €1000 [65, 66]. We allow room here for a more positive outcome in costs, as we predict an average cost saving per participant of €1113.75 for enhanced therapy based on the expected clinical effect of 59% relative reduction in number of ulcers compared with usual care (see Table 1). With a willingness to pay (WTP) of €50,000, a correlation between costs and QALYs (*ρ*) of 0, *α* = 0.05 (two-sided), power = 80%, and a drop-out rate of 20%, this leads to a sample size of 63 participants per study group, or 126 participants in total. Without drop-out, a sample size of (63 × 0.8 =) 51 participants per study group or 102 in total is required.

**Table 1:** Average predicted costs per participant

				**Enhanced therapy**		**Usual care**	
**Modality**	**Event/activity**	**Time (hours)**	**Unit costs**	**Expected frequency**	**Expected costs**	**Expected frequency**	**Expected costs**
	Ulcer development [6]	-	€10,000	0.15	€1500	0.37	€3700
	Podiatrist	1	€100	0.50	€50	0.00	€0
Custom-made footwear	Optimization equipment: pressure measurements [36]	-	€50	1.00	€50	0.00	€0
	Shoe technician	1	€100	2.00	€200	0.00	€0
Custom-made indoor footwear	Production and optimization [13]	-	€400	0.95	€380	0.00	€0
Personalized temperature monitoring	Thermometry [38]	-	€75	0.75	€56.25	0.00	€0
Personalized education	Structured education	1	€100	0.50	€50	0.00	€0
	Motivational Interviewing	1	€100	2.00	€200	0.00	€0
	Equipment [49, 57]	-	€100	1.00	€100	0.00	€0
**Total**					€2,586.25		€3,700

Average of predicted additional costs per participant of enhanced therapy versus usual care, based on the expected frequencies as described in the power calculation. For all costs except ulcer development, usual care is used as index. Unit cost estimations are based on previous studies and standard hourly wages of a college educated professional in the Netherlands

With the power calculations showing a requirement of 122 participants for the primary clinical outcome, 120 for the primary patient-related outcome, and 126 for the primary health-economic outcome, a sample size of 126 participants provides adequate power for all three primary outcomes.

### Recruitment {15}

Patients will be recruited both retrospectively from outpatient visit schedules and prospectively. Recruitment takes place at all participating centers, i.e., Amsterdam UMC (location AMC and location VUmc), Máxima Medisch Centrum (Veldhoven), and Reinier de Graaf Gasthuis (Delft) in the Netherlands. Recruitment will continue until a total of 126 participants have been included and randomized into the study. We anticipate this to take 18 months to complete. Each patient will be followed for 12 months for ulcer development and other outcomes, and for an additional 6 months for ulcer and cost-utility outcomes if an ulcer is present at 12 months’ follow-up.

### Assignment of interventions: allocation

#### Sequence generation {16a}

Participants will be randomly assigned to either enhanced therapy or usual care using an independent method, being an online-accessible computer program (Castor EDC, Amsterdam, the Netherlands) that uses a variable block randomization method. Randomization will be stratified according to participating center (with both locations of Amsterdam UMC seen as 1 center) and time since healing of last ulcer with a gender-specific cut-off (6 months for men and 10 months for women, based on median values of time since healing of last ulcer in a previous study [20]).

#### Concealment mechanism {16b}

The allocation is computer-generated at the moment of randomization using an independently-generated sequence (Castor EDC, Amsterdam, the Netherlands) and therefore concealed until the moment of randomization.

#### Implementation {16c}

The code for allocation sequence will be prepared by a non-involved investigator. Once written, the code is stored in Castor EDC.

### Assignment of interventions: blinding

#### Who will be blinded {17a}

The outcome assessors of the primary clinical outcome (i.e., ulcer or no-ulcer) will be blinded to group allocation. Participants, shoe technicians, and investigators cannot be blinded to group allocation. We aim to blind the involved physicians and podiatrists by not informing them about group allocation; however, because the participant and shoe technician are not blinded, blinding of the involved physicians and podiatrists cannot be guaranteed. Primary statistical analyses will be done by an investigator blinded to group allocation.

#### Procedure for unblinding if needed {17b}

Both the investigator and the participant are not blinded and therefore no procedures for unblinding them are required. Involved physicians and podiatrists can be notified about group allocation by the investigator when deemed necessary. The investigator performing the primary statistical analyses will be unblinded after the analyses are conducted.

### Data collection and management

#### Plans for assessment and collection of outcomes {18a}

The study investigators will perform all study measurements, during baseline, randomization, and follow-up visits. The treating podiatrist or physician will initially assess foot ulcer outcomes (when occurring), including taking photographs of the foot. See Fig. 2 for an overview of the study procedures.

##### Baseline assessments to confirm definitive eligibility

If an eligible participant consents to participate, they will undergo a baseline assessment at the participating hospital to confirm eligibility for inclusion in the study. The following characteristics will be obtained during the baseline visit:

- Demographic information and disease-related characteristics (e.g., diabetes duration and control, presence of complications, ulcer history, footwear use, etc.).
- Peripheral neuropathy assessment, consisting of testing for the presence of neuropathy by measuring the loss of protective sensation by using the 10-g (5.07) Semmes Weinstein monofilament at the plantar surface of the hallux and the first and fifth metatarsal heads of both feet, and with a 128-Hz Tuning fork held on the apex of the great toe [31].
- Peripheral arterial assessment by palpation of the dorsalis pedis and posterior tibial pulses in both extremities, according to the wound, ischemia, and foot infection (WIfI) classification system [60, 61]. Additional vascular assessment will be done by measuring the toe-pressure [60, 61].
- Presence of foot deformity will be assessed clinically. These include hammer/claw toes, prominent metatarsal heads, hallux valgus, pes planus, pes cavus, Charcot deformity, and any amputation. Participant’s feet will be classified into one of four categories according to the severity of deformity present: no deformity, mild deformity, moderate deformity, and severe deformity [10].

##### Assessments at baseline visit

If eligibility has been confirmed, photographs of the plantar and dorsal surfaces of both feet will be taken according to a standardized protocol [10] and foot-related self-care behavior will be assessed using a 7-item questionnaire based on a commonly used questionnaire by the Dutch Society of Podiatry (Nederlandse Vereniging voor Podotherapie, NVvP) [67]. Quality of life will be assessed by using the SF-36 and EQ-5D-5L questionnaires [5, 46]. Participants will complete a study questionnaire consisting of the following topics: satisfaction with ulcer prevention treatment; use and usability of their custom-made footwear (using a study-specified Monitor Orthopedic Footwear [51]); capabilities, opportunities, and motivations for ulcer prevention using an adapted COM-b questionnaire [54]; and knowledge of foot care (questions 24–29 from the Patient Interpretation of Neuropathy questionnaire [53]).

Furthermore, barefoot plantar pressure distribution during standing and walking will be measured using the Novel EMED-X platform system (Novel GmbH, Munich, Germany), which consists of pressure-sensing sensors (4 per cm^2^) that are sampled at 50 Hz. Four trials of both feet at the subjects’ self-chosen comfortable pace using a “two-step” gait approach to the platform will be collected [68]. In-shoe plantar pressure distribution will be measured inside the two most frequently used pairs of custom-made footwear. In-shoe pressures will be measured with the Pedar-X system (Novel GmbH, Munich, Germany), which comprises flexible pressure-sensing insoles (99 sensors, 50Hz sampling rate) placed between the sock and the shoe insole. Measurements will be performed at a self-chosen comfortable speed along a walkway of at least 10 m; gait speed will be recorded and kept within 10% for all walking trials. A minimum of 12 mid-gait steps per foot will be recorded [69].

Additionally, physical activity and wearing time of the custom-made footwear will be assessed. Physical activity will be measured for seven consecutive days following the baseline visit using a MoveMonitor (McRoberts, the Hague, the Netherlands), worn in a belt around the middle at vertebrae L5 [57]. Participants also receive a short diary for these 7 days to keep track of time points and activities throughout the week. All custom-made footwear will be equipped with a validated temperature sensor in a cavity created underneath the top layer of the shoe insert (OrthoTimer; Rollerwerk, Balingen, Germany) [49]. This will be used to objectively assess footwear use by the participant throughout the trial.

##### Assessments at randomization

Two weeks after study entry, the randomization visit will take place at the orthopedic shoe company. The feet of the participant will be checked for foot ulceration. If a participant has a foot ulcer, they will be immediately referred to the multidisciplinary team, and randomization will be postponed until the ulcer is healed for 2 weeks. If there is no foot ulcer, and all other eligibility criteria are also still met, the participant will be randomized to one of the two study groups.

##### Assessments during follow-up

Each participant will be followed for 12 months. If a participant has a foot ulcer at 12 months, they will be followed for another 6 months or until healing, whichever comes first. Follow-up will take place after 3, 6, 9, and 12 months (FU3, FU5, FU7, and Final visit (FV), respectively). Prior to each regular study visit, all participants will receive the EQ-5D-5L questionnaire and study specified versions of the iMTA iMCQ and iMTA iPCQ to gather volume data on medical costs, family costs, and productivity loss [45]. Additionally, at 6 and 12 months, all participants also complete the study questionnaire (see the section “Assessments at baseline visit”), and at 12 months also the SF-36. Participants in enhanced therapy will hand in their weekly diaries.

Custom-made footwear wearing time will be assessed during each 3-monthly visit by reading out the OrthoTimer sensor in the shoe. At 6 and 12 months, physical activity is assessed by wearing the activity monitor for 1 week. At 12 months, barefoot and in-shoe plantar pressure measurements will be repeated. Use of intramural healthcare resources during the study will be obtained at final visit from the participants’ medical status. If a participant has a foot ulcer at 12 months, visits will be scheduled at 15 and 18 months, or 2 weeks after healing (whichever comes first). During these visits, volume data on medical costs, family costs, and productivity loss will be gathered (iMTA) and the EQ-5D-5L will be completed. An overview of all measurements can be seen in Fig. 2.

##### Unscheduled assessments: foot ulceration

If the participant, treating physician, or podiatrist identifies an ulcer in-between 3-monthly regular visits, they are instructed to inform the investigator immediately, and have photographs taken of the foot by the treating physician or podiatrist. The podiatrist will debride the wound first if required to assess outcome, and will classify the ulcer using the University of Texas Wound Classification [59] and the WIfI-score [60, 61]. This information will be sent to the investigator, who will upload all information anonymously to a web-based environment for ulcer outcome assessment. A panel of blinded and independently operating physicians with > 10 years of experience in diabetic foot care will then assess these photographs. Participants will complete the validated Dutch version of the Cardiff Wound Impact Schedule [55, 56] 4 and 12 weeks after an ulcer occurred, provided the ulcer has not yet healed. Participants will complete the EQ-5D-5L one extra time, 4 weeks after an ulcer occurred, as presence of a foot ulcer is known to reduce quality of life [70]. Participants will then continue with the follow-up assessments as scheduled.

#### Plans to promote participant retention and complete follow-up {18b}

Participant retention is promoted by regular contact with the participant. A reminder of an upcoming visit will be sent by e-mail or via a phone call, depending on the participant’s preference. Furthermore, study visits will, whenever possible, be scheduled on the same day of a regular clinic visit to minimize the number of extra visits. If a participant withdraws from the study, information on ulcer outcome at 12 months will be obtained from the participant’s medical files, if a participant consents to this procedure. For participants who do not consent, ulcer outcome will be based on outcome at the moment of withdrawal (last observation carried forward). Ulcer outcome data from participants who die during the study will be based on outcome at the moment of death (last observation carried forward).

#### Data management {19}

A complete data management plan has been composed in collaboration with experts in data management of Amsterdam UMC. Data will primarily be collected on paper using case report forms specifically designed for this study and each visit, and saved in an electronic clinical data management platform compliant with all legal and ethical rules (Castor EDC, Amsterdam, the Netherlands), and with restricted range checks on data values. Diaries and questionnaires are completed on paper and saved in the database upon receipt, or completed digitally. Measured data of the Pedar-X and EMED-X systems (Novel GmbH, Munich, Germany) is stored on network drives of Amsterdam UMC, using standardized operating procedures. Raw data from activity monitors (MoveMonitor, McRoberts, the Hague, the Netherlands) and adherence monitors (Orthotimer, Rollerwerk, Balingen, Germany) is stored on the approved servers of the companies and transferred for analysis to Amsterdam UMC network drives using standardized operating procedures. Contractual agreements are in place for the use of measured data from these external parties.

#### Confidentiality {27}

Participants will be coded by the abbreviation of the participating center combined with a 3-digit participant number. All data related to patients will be safely stored in a locked office cabinet or on a password-protected computer file. Only involved researchers will have access to this study information. Participant’s name will only be recorded on the informed consent forms, which will be kept in a locked cabinet with the lead investigator per center, separately from study data. The subject identification log will be kept separate from all other study related datasets, to assure there is no possibility to match study data with identifiable personal data. All study information will be saved for at least 15 years after the study has ended.

#### Plans for collection, laboratory evaluation and storage of biological specimens for genetic or molecular analysis in this trial/future use {33}

Not applicable. No biological specimens for genetic of molecular analysis will be collected for this study.

### Statistical methods

#### Statistical methods for primary and secondary outcomes {20a}

Statistical analysis will be performed after the last follow-up visit of the last participant using SPSS statistical software (IBM Corporation, Armonk, NY). All tests will assess group effects, will be two-sided, and use *P* < 0.05 as significance level. All comparisons between groups are based on both intention-to-treat and final visit end-point analysis.

##### Primary study outcomes

For each of the three specified outcomes related to proportion of participants with a recurrent foot ulcer (any ulcer, plantar ulcer, ulcer at high-risk location), effectiveness of the intervention will be assessed using chi-square analysis. Outcome of ulcer recurrence over time will be assessed using log-rank testing and presented as Kaplan–Meier plots censored for death. For adherence to wearing custom-made footwear, Student’s *T*-test for independent samples will be done, provided the data is normally distributed. Cost and utility outcomes will be combined in the economic evaluation to calculate cost (savings) per QALY (gained) per participant (i.e., cost-utility). Incremental cost-utility ratios (ICUR) will be calculated as the ratio between costs and QALY differences between enhanced therapy and usual care. To account for sampling variability, non-parametric bootstrapping stratified for group and participating center category will be done with 5000 replications. Results will be displayed graphically by plotting the bootstrapped differences in costs and QALYs in a cost-utility plane, with the percentage of pairs in each quadrant calculated. Finally, cost-utility acceptability curves (CUAC) for willingness to pay values up to €100,000 will be graphically displayed, with the probability of enhanced therapy being cost-effective over usual care calculated for willingness-to-pay levels of €0,- and €50,000 per QALY gained and €0,- and €20,000 per extra participant without foot ulcer recurrence, respectively.

##### Other study outcomes

Differences in costs and QALYs between enhanced therapy and usual care will be assessed using independent samples *T*-tests, with 95% bias-corrected and accelerated confidence intervals around the mean difference to account for sampling variability, stratified for a maximum of two variables that are univariately associated with the outcome. For cost-effectiveness, the approach will be identical to the approach for cost-utility described above, with foot ulcer recurrence as the clinical outcome. For all other study parameters (see the “Outcomes {12}” section), differences between enhanced therapy and usual care will be assessed via independent sample* T*-tests (for normally distributed data) or Mann–Whitney *U*-tests (for not normally distributed data) in case of interval or ratio data, and with chi-square or Fisher’s exact tests in case of nominal or ordinal data.

In addition, a budget impact analysis (BIA) will be done. The budget impact analysis will be carried out from governmental, health care provider, and insurer perspectives. The governmental perspective is chosen to help setting priorities in health care optimization while simultaneously considering the wider implications of stimulating enhanced therapy for diabetic patients at a high risk of ulcers beyond the health care sector. The provider perspective is chosen to support local decisions on economies of scale and affordability. The insurer perspective is chosen to assess the net financial consequences of offering intensified monitoring to high-risk patients who already have had ulcers in the past, which may help to shift health care use from the second to the first echelon. The BIA will be conducted using a decision-tree model developed in Microsoft® Excel. The BIA will be performed according to the Professional Society for Health Economics and Outcomes Research Task Force principles. We will use multiple scenarios with different numbers for the population to target, different treatment adherence, and different implementation scenarios, based on the best estimates available at the time of analysis.

#### Interim analyses {21b}

Not applicable. This trial will not have an interim analysis.

#### Methods for additional analyses (e.g., subgroup analyses) {20b}

Various per-protocol analyses will be done, consisting of (i) adherence to the modalities in enhanced therapy and (ii) adherence to each specific modality in enhanced therapy, comparing adherent participants with both non-adherent participants and with the control group, and (iii) for each modality that is part of the intervention, we will compare outcomes of all participants who received that modality (including those in usual care, since the separate parts of the intervention can be provided in usual care) with participants who did not receive that modality.

Subgroup analyses will be done based on gender, ethnicity, disease severity (e.g., ulcer history, comorbidities), education level, and disease perceptions (e.g., perceived ulcer risk), to assess their associations with all outcomes.

#### Methods in analysis to handle protocol non-adherence and any statistical methods to handle missing data {20c}

As the first choice, missing data will be imputed using multiple imputation with a fully conditional specific predictive mean matching model. Baseline characteristics that significantly differ between those with complete and those with missing data will be used as predictors. For sensitivity analysis, other imputation strategies (e.g., with fewer predictors, or single imputation strategies using individual or group means) will be applied as well.

#### Plans to give access to the full protocol, participant-level data and statistical code {31c}

Public access can be granted to the full study protocol, syntaxes or scripts, and the data dictionary once the study is completed, following reasonable request to the investigators. Furthermore, raw, preprocessed, and processed data essential to check study conclusions will be published in the digital archive Figshare once the outcomes are published.

### Oversight and monitoring

#### Composition of the coordinating center and trial steering committee {5d}

There will be multiple investigators of the coordinating center involved in the day-to-day support for the trial. They will meet to discuss matters associated with the trial on a regular basis and when needed.

#### Composition of the data monitoring committee, its role and reporting structure {21a}

The study will be monitored by the independent Clinical Monitoring Center (CMC) of Amsterdam UMC. Monitoring tasks that apply for this study are the enrolment progress, informed consent procedure, source data review and verification, safety reporting, and completeness of the trial master file and investigator site files. After every monitoring visit, a site monitoring visit report is written that provides an overview of all monitoring issues, findings, or discrepancies. The CMC clinical research associates involved in the monitoring process are independent from the study and study team and have no competing interests.

#### Adverse event reporting and harms {22}

All adverse events (AEs) reported spontaneously by the participant, their treating clinicians, or observed by the investigators will be recorded using a case report form (CRF). Serious adverse events (SAEs) will be reported to the sponsor without undue delay after obtaining knowledge of the event by the investigator. The sponsor will report the SAEs to the accredited Medical research ethics committee (METC) that approved the protocol, within 7 days of first knowledge for SAEs that result in death or are life-threatening followed by a period of a maximum of 8 days to complete the initial preliminary report. All other SAEs will be reported within a period of a maximum 15 days after the sponsor has first knowledge of the SAE.

#### Frequency and plans for auditing trial conduct {23}

Given the monitoring plan, no audits are planned.

#### Plans for communicating important protocol amendments to relevant parties (e.g., trial participants, ethical committees) {25}

All amendments to the protocol will be reported to and assessed by the Medical Ethics Review Committee of Amsterdam UMC. Participants will be notified when amendments are substantial according to the Medical Ethics Review Committee.

#### Dissemination plans {31a}

All outcomes will be published in peer-reviewed publications and presented at scientific conferences. Associated healthcare professionals and companies will be informed about the results as soon as possible after finishing the trial. Furthermore, an implementation committee consisting of patient representatives, healthcare professionals, and an implementation expert is already involved in the trial to discuss and prepare implementation strategies after the trial results are obtained.

## Discussion

The DIASSIST trial aims to assess the effect of an integrated personalized assistive devices approach aimed to help prevent foot ulcer recurrence in people with diabetes on effectiveness, treatment adherence, and cost-utility. We designed this intervention and this RCT using a multidisciplinary systematic design approach, that consisted of the following steps: (i) systematic literature search and assessment of available evidence; (ii) assessment of clinical guidelines; (iii) first design of the intervention; (iv) multidisciplinary discussions with patients, healthcare professionals, and researchers; (v) testing the design with patients; and (vi) final design of the intervention. In this discussion, we will describe the considerations and outcomes of these steps.

### (i and ii) Assessment of evidence and guidelines

We systematically searched the literature for proven interventions that help prevent diabetic foot ulceration. We identified the four modalities as described in the “Interventions” section to be relevant to include in our design process. Results of these previous studies indicated a positive effect of the interventions, especially when treatment adherence is high [10, 14, 15, 21]. In addition to the 29 publications about single modalities as referenced in the “Interventions” section, we identified three studies that assessed a form of integrated care [71–73]. However, none combined all the abovementioned single interventions. We built upon the concept of integrated care from these studies by combining multiple interventions and expanded this concept by combining more and different modalities and using a personalized approach.

We subsequently assessed international clinical guidelines on current recommendations for the prevention of plantar foot ulcer recurrence [7]. Three modalities, i.e., custom-made footwear, daily at-home monitoring of foot temperatures, and patient and family education, are included in the guidelines, but these are not, or not according to a state-of-the-art protocol, implemented in clinical practice. Custom-made footwear specifically for indoor use is a new treatment, and as such not recommended in guidelines [7]. In addition, although there is no scientific evidence yet, there is a strong recommendation in the guidelines to integrate these treatments.

### (iii–v) First design, multidisciplinary discussions, and pilot testing

Combining the outcomes from previous trials and the guideline recommendations with our clinical experience, we designed the first version of our state-of-the-art integrated approach. The approach is designed to assess patients who are at high risk for developing a plantar foot ulcer and who are expected to benefit from the combined modalities. We therefore decide to exclude patients who have had an ulcer on the apex of their toes as the only ulcer location in the last 4 years up until inclusion. For these patients, a flexor tendon tenotomy would likely be a better first treatment option than the combination offered in this trial [7, 30]. Patients with a bilateral amputation proximal to the Chopart joint are also excluded, since the primary working mechanism of the intervention is aimed at reducing plantar foot ulcer recurrence by optimizing footwear and footwear adherence, and both are not applicable to these patients.

The first design of the intervention was then discussed during multiple meetings with a total of 22 experts from different disciplines. This included specialists from rehabilitation and internal medicine, shoe technicians, podiatrists, researchers, and patient representatives. In addition, we discussed the design with an independent advisory board of seven members representing a variety of expertise, including internal medicine and vascular surgeon specialists, representatives of the Dutch podiatry association (NVvP), a patient representative from the Dutch Diabetes Association (DVN), and an implementation expert of Amsterdam UMC. This committee will also continue to meet on a yearly basis to be informed about trial progress, and to discuss future implementation. Given the extensive involvement of multiple stakeholders, we expect that implementation of the integrated and personalized approach will be facilitated, provided it is successful.

The approach was tested in three people with diabetes at high risk of ulceration, who were given the opportunity to provide feedback on the design and their experiences. Testing included the production of custom-made indoor footwear, the patient education model, and the trial visit procedures. Based on the discussions with experts, the implementation committee, and patient representatives, minor adjustments were made before finalizing the design. These adjustments were mainly related to the visual feedback and communication towards participants using the personalized FFTT model. We also adapted the first visit for patient education after the evaluation of the first participants in the RCT. This visit was originally scheduled 6 weeks after randomization, to provide feedback on footwear adherence as measured at baseline. However, the indoor footwear was provided in that first 6-week period, which made the feedback on treatment adherence outdated, resulting in confusion with the participants. The first visit for patient education was therefore rescheduled to 1 week after randomization, as described in the “Participant timeline {13}” section.

### (vi) Final design

The final design of the integrated personalized assistive devices approach to help prevent foot ulcer recurrence in people with diabetes is as described in the “Interventions” section.

### Strengths and limitations

A strength of this trial is being powered for three primary outcomes, including a clinical outcome (foot ulcer recurrence) generally used in foot ulcer prevention trials [15], a patient-related outcome (footwear adherence) that captures the patient’s perspective, and a health-economic outcome (cost-utility). This combination of perspectives is a major strength, as it can offer evidence in multiple directions. Especially including cost-utility as one of the primary outcome measures is a strength, as this is hardly investigated in this field, despite frequent calls for insights into health-economy [7]. Since personalization requires more time from healthcare professionals, the intervention will incur extra costs. Furthermore, the use of assistive devices and sensors also increases costs. It is therefore necessary to investigate whether the benefits outweigh the extra costs. Both cost-utility and cost-effectiveness are included as outcomes in this trial. Cost-utility has been chosen over cost-effectiveness because of its generalizability, as QALYs are a universal measure for which the willingness to pay can be determined independent of the patient population [45]. Cost-utility is also used as the primary outcome for the sample size calculation, which deviates from how most clinical studies conduct a sample size calculation, taking ulceration as primary outcome. We chose cost-utility, as this may be seen as a more important outcome than ulceration alone, because it takes the quality of life of participants and the costs into account, with changes in ulcer outcome being negotiated in these personal and health economic outcomes. Another strength is the focus on a personalized and state-of-the-art approach, to use the latest evidence on effective treatment and to consider this treatment with respect to usability and effectiveness for the individual patient, thereby reducing patient burden and increasing treatment adherence where possible.

A potential limitation in this trial is the multiplicity and diversity of treatments offered. Our main goal is to offer a combined treatment approach that is representative of a combination of treatments that patients can receive in usual care, but personalized and optimized in a structured fashion. While this is a strength from the perspective of generalizability, it may not be possible to determine the individual contribution of the modalities to the overall effect. Another limitation is that all interventions offered can currently be part of usual care, which, if the case, may mask an effect between enhanced therapy and usual care. Although it is known that these interventions are not widely implemented in Dutch healthcare for diabetic foot disease [27], this is a potential threat to group contrast. However, by conducting a per-protocol analysis for both the combined interventions and for each specific modality, we aim to gain further insights into the effects of the individual treatment modalities. This analysis will include participants of the control group who receive one of the enhanced therapy interventions as part of usual care.

## Conclusion

In conclusion, the DIASSIST trial aims to provide evidence for the effect of an integrated personalized assistive devices approach to help prevent foot ulcer recurrence in people with diabetes. The trial primarily assesses the effectiveness, footwear adherence, and cost-utility, to investigate three different perspectives for which the trial is adequately powered: a clinical, a patient-related, and a health-economic perspective, respectively. This is the first RCT to investigate a combination of treatments for ulcer prevention, tailored towards the individual patient, with clinical and economic outcome analyses. When proven effective, this approach can be implemented in healthcare and improve preventative care for people with diabetes at high risk of foot ulceration and with that improve quality of life and reduce healthcare costs.

## Trial status

This trial was registered on clinicaltrials.gov in February 2022, ID: NCT05236660. The trial commenced recruitment in February 2022 and recruitment is expected to be completed in December 2023. Data collection for all enrolled subjects will continue through December 2024, with the possibility of extension through July 2025 when a foot ulcer is present in participants at the end of follow-up.

### Supplementary Information

**Additional file 1.**

## Data Availability

The investigators have access to all data and materials. The data collection can be found through an online repository, i.e., Figshare. Availability and reuse of the data are covered in the informed consent procedure.

## Abbreviations

(S)AE: (Serious) adverse event
BASE: Baseline visit
BIA: Budget impact analysis
Castor EDC: Electronic Data Capture system
CMC: Clinical Monitoring Center
COM-b: Capabilities, Opportunities and Motivations questionnaire
CRF: Case report form
CUAC: Cost-utility acceptability curves
EQ-5D-5L: Quality-adjusted life years questionnaire
FU: Follow-up visit
FV: Final visit
ICUR: Incremental cost-utility ratios
iMCQ iMTA: Medical Consumption Questionnaire
iMTA: Institute for Medical Technology Assessment
iPCQ iMTA: Productivity Cost Questionnaire
METC: Medical research ethics committee, in Dutch: *Medisch Ethische Toetsings Commissie*
NVvP: Dutch Society for Podiatry, in Dutch: *Nederlandse Vereniging voor Podotherapie*
OSA: Orthopedic footwear type A
OSB: Orthopedic footwear type B
OVAC: Orthopedic Provision in Regular Footwear
PEDIS classification: Perfusion, Extent, Depth, Infection, and Sensation classification
PIN: Patients’ Interpretation of Neuropathy questionnaire
QALY: Quality-adjusted life year
RAND: Randomization visit
RCT: Randomized controlled trial
SF-36: Quality of life questionnaire
SPIRIT: Standard Protocol Items: Recommendations for Interventional Trials
SPSS: Statistical Package for the Social Sciences, statistical software platform
UV: Unscheduled visit
WIfI-score: Risk stratification score based on Wound, Ischemia, and Foot Infection
WMO: Medical Research Involving Human Subjects Act, in Dutch: Wet Medisch-wetenschappelijk Onderzoek met Mensen
WTP: Willingness to pay
